# The Mediating Role of Stress in the Relationship Between Self-Efficacy and Medication Adherence Among Patients with Heart Failure: A Cross-Sectional Study in the Aseer Region, Saudi Arabia

**DOI:** 10.3390/healthcare14040462

**Published:** 2026-02-12

**Authors:** Lizy Sonia Benjamin, Mesheil Alalyani, Richard Maestrado, Amal Naif Alreshidi, Majid Ali Alotni, Nuha Ayad H. Alatawi, Amutha Chellathurai, Analita Gonzales, Allen Joshua Dominguez, Kawther Eltayeb Ahmed, Sabah Abdullah Mohammed Asiri

**Affiliations:** 1 Medical-Surgical Nursing Department, College of Nursing, King Khalid University, Abha 62521, Saudi Arabia; 2 Department of Adult and Advanced Nursing Care, College of Nursing, King Khalid University, Khamis 61421, Saudi Arabia; 3 Department of Medical-Surgical Nursing, University of Hail, Hail 55471, Saudi Arabia; 4 Maternity and Children Hospital, Hail Health Cluster, Hail 55471, Saudi Arabia; 5 Department of Medical-Surgical Nursing, College of Nursing, Qassim University, Buraydah 51452, Saudi Arabia; 6 Medical-Surgical Department, Faculty of Nursing, University of Tabuk, Tabuk City 71491, Saudi Arabia; 7 Saveetha College of Nursing, Saveetha University, Velappanchavadi, Chennai 600077, Tamilnadu, India; 8 Nursing Administration and Education Department, Faculty of Nursing, University of Tabuk, Tabuk City 71491, Saudi Arabia; 9 College of Nursing, Shaqra University, Riyadh 11911, Saudi Arabia; adominguez@su.edu.sa; 10 Fundamental of Nursing Department, College of Nursing, King Khalid University, Abha 62521, Saudi Arabia; 11 Aseer Central Hospital, Ministry of Health, Abha 62523, Saudi Arabia

**Keywords:** cardiac problems, medication adherence, adherence, self-efficacy, stress, psychological

## Abstract

**Introduction**: Understanding how self-efficacy relates to medication adherence is crucial for patients with heart failure. This study investigated how stress mediates the relationship between self-efficacy and adherence to prescribed medication in patients with heart failure. **Methods**: This study employed a cross-sectional descriptive design. Using convenience sampling, 270 participants were recruited from the outpatient cardiology clinic in one of the largest hospitals in Aseer region, Saudi Arabia. Participants completed self-administered questionnaires, including the Perceived Stress Scale (PSS), Cardiac Self-Efficacy Scale (CSES), and Adherence to Refills and Medications Scale (ARMS). The data collection was conducted from January 2025 and ended in May 2025. **Results**: Results showed moderate stress (Mean = 20.17), high self-efficacy (Mean = 44.90), and a tendency toward medication non-adherence (Mean = 23.38). Stress was positively correlated with medication non-adherence (r = 0.392, *p* < 0.01), while self-efficacy was the strongest predictor of adherence (β = −0.345, *p* < 0.001). Mediation analysis confirmed that perceived stress partially mediates the relationship between self-efficacy and medication adherence (indirect effect β = −0.063, *p* = 0.003). **Conclusions**: Higher self-efficacy reduces perceived stress, which in turn leads to better medication adherence. From a nursing perspective, clinical practice should shift toward a “confidence-based” model of care. Routine psychological screening and targeted, demographic-specific interventions—particularly for younger patients and those with multiple comorbidities—are essential to empower patients and improve long-term health outcomes.

## 1. Introduction

Self-efficacy is defined as the degree of confidence a person has about their capacity to implement the actions required to accomplish a desired task or achieve a specific outcome [1]. For heart failure patients, self-efficacy is a critical determinant of whether they adhere to complex medication frequencies and dosages [2,3]. Existing research emphasizes that self-efficacy does not operate in isolation; it acts as a critical mediator between social support and adherence [4,5], and links patient education to broader self-care behaviors [6,7]. While educational programs can enhance self-efficacy and increase the likelihood of medication adherence [8], the literature suggests that knowledge alone is insufficient—proactive self-care only occurs when an individual believes they possess the capability to act [9].

Furthermore, stress significantly impacts this dynamic, negatively affecting medication regimens for individuals with low self-efficacy or depression [10,11]. While self-efficacy acts as a protective factor against the negative effects of stress on adherence [12], evidence indicates that stress can conversely deplete an individual’s self-efficacy, creating a cycle of reduced adherence [12]. Based on Bandura’s Social Cognitive Theory (SCT), health behaviors are a product of the interaction between personal beliefs (self-efficacy) and social/environmental stressors [2,13,14,15]. While interventions targeting self-efficacy have produced clinically significant gains in adherence rates [2], there is a critical need to understand how psychological barriers—specifically stress—interfere with this relationship.

Despite the established links between self-efficacy and adherence, there is a gap in understanding the specific mechanism through which psychological stress influences this path in heart failure patients within the Saudi Arabian context. This study addresses this problem by examining whether stress acts as a bridge or a barrier between a patient’s confidence and their actual medication-taking behavior. In evaluating these intersecting psychosocial factors, the study advocates for a shift toward comprehensive mental health screening and stress management within heart failure protocols. Consequently, the primary aim of this study was to determine the mediating effect of stress on the relationship between self-efficacy and medication adherence among individuals diagnosed with heart failure.

## 2. Methods

### 2.1. Design

A cross-sectional descriptive design was employed using a self-report questionnaire in this study.

### 2.2. Sampling/Setting

The study took place at the outpatient cardiology clinic of Aseer Central Hospital in Abha, Saudi Arabia. Aseer Central Hospital is one of the largest tertiary and teaching hospitals in the country and provides all levels of care to patients with a focus on cardiology; the majority of the referrals to the Aseer region are sent there for management of complex cases. The large number of cardiac cases managed and its specialized cardiology services made it an ideal location to recruit the desired participant population.

Participants were recruited by using a convenience sampling methodology. The target population consisted of adults (18 years) who were diagnosed with heart failure that was classified as NYHA (New York Heart Association) Functional Classes II–IV. Eligible patients included those who had been on a consistent and stable heart failure medication regimen for at least three months and could provide informed consent. Excluded from participation were patients who were critically ill and had a history of cognitive impairments or psychiatric illnesses based on patient self-reports during the initial screening interview. The target number of participants needed for this study was calculated using Cochran’s formula for sample size determination in cross-sectional studies: n = Z^2^ P (1 − P)/d^2^; where n is the required sample size; Z is the Z-statistic for the 95% confidence level (1.96); P is the expected prevalence or proportion (0.50 used to maximize variance); d is the precision or margin of error (0.05). Based on these parameters, the calculation (1.96^2^ × 0.5 × 0.5/0.05^2^) yielded a requirement of 384; however, the target recruitment goal was adjusted to 270 participants based on finite population correction or specific power analysis for mediation.

### 2.3. Questionnaire

The demographic profile was developed based on established predictors of cardiac health outcomes in similar populations [16]. Variables include age, gender, marital status, educational level, employment status, comorbidities, and duration of illness (years). These factors were selected to control for potential confounding effects on stress and medication adherence. The three questionnaires were in English language as the patients understand English.

The PSS-10 was selected for its global validity in assessing psychological distress across various clinical populations, providing a broader perspective on the patient’s stress levels compared to disease-specific measures. Scores are obtained by reversing the responses to the four positive items (Items 4, 5, 7, and 8) from 0 = 4, 1 = 3, 2 = 2, 3 = 1, and 4 = 0, and then summing all 10 items. Total scores range from 0 to 40. Scores are interpreted as: 0–13 (low stress), 14–26 (moderate stress), and 27–40 (high stress) [17]. These categories allow for the stratification of patients during correlational analysis.

The CSES was chosen due to its specific focus on the self-regulatory behaviors required for cardiac recovery, which generalized self-efficacy scales lack. The instrument consists of 13 items on a 5-point Likert scale (0–4). The total score is the sum of all items, ranging from 0 to 52. Higher scores indicate greater confidence in managing heart health. For this study, scores were treated as a continuous variable; however, a score of 52 represents the ceiling of “very confident” across all domains [18].

The ARMS is used to evaluate medication adherence. Crucially, the ARMS utilizes a reverse-scoring interpretation: lower scores represent higher adherence. Items are rated from 1 (none of the time) to 4 (all of the time). The total score is the sum of the 12 items (range 12–48). A score of 12 indicates “perfect adherence,” while higher scores signify increasingly poor adherence [19]. This inverse relationship was strictly maintained during data entry to ensure the integrity of the correlation with PSS and CSES scores.

Content validity was established through a panel of four experts (two cardiologists, one cardiac nurse, and one psychometrician). To quantify this process, the Content Validity Index (CVI) was calculated. The panel achieved an I-CVI of 1.00 for all items, exceeding the recommended threshold of 0.78 for four or more experts. The instruments have demonstrated strong internal consistency in previous studies, with Cronbach’s alpha of 0.90 for the PSS [20], 0.90 and 0.87 for CSES [18] (for Control Symptoms and Maintain Functioning, respectively, ref. [18] and 0.81 for the ARMS [19]. In the current pilot study (N = 20, not included in the final sample), Cronbach’s alpha was 0.83 (PSS), 0.80 and 0.98 for Control Symptoms and Maintain Functioning respectively for CSES, and 0.78 (ARMS), confirming their reliability in this specific context.

### 2.4. Data Collection Methods

Trained clinical staff were responsible for identifying participants. Once participants had been identified, researchers were then responsible for approaching them individually. Researchers ensured that participants understood the purpose of each question via a “teach back” method and provided them with printed instruction sheets. Due to the demographics of cardiology patients, a paper and pencil self-administered survey was used in the study. Therefore, researchers provided participants with an informed consent form which participants could complete at their own leisure and return with them at a later date at one of their scheduled clinic appointments. Follow-up reminders during future clinic appointments were made available to participants to help prevent potential biases in participant attrition resulting from the delayed return method. Additionally, researchers maintained a tracking log to monitor the return of surveys as well as the lead investigators periodically monitored to ensure consistent implementation of the “teach back” method and the continued protection of confidentiality and anonymity of participants throughout the study.

### 2.5. Ethical Considerations

This study has with the approval of the Institutional Review Board of King Khalid University (ECM# 2024–3169, dated 9 December 2024). The researchers took several foundational measures to protect the participant’s well-being and to protect the scientific integrity of the study as they ensured the research met the established nursing research standards. All participation in the study was completely voluntary, and during the recruitment process, the researchers used a “teach-back” method to ensure that the participant did not simply sign an informed consent form but also understood what they had agreed to. Researchers protected the participants’ confidentiality and privacy by using unique codes to protect their identities throughout the data collection and then storing physical copies of the data in secured locked facilities that could be accessed only by the research team. In addition, the design of the study gave priority to non-maleficence (do no harm) by allowing participants to collect their data on their own time and therefore reducing participants’ stress and not disrupting their clinical treatment. A valid and reliable instrument was chosen for the research tool because it is a widely accepted and publicly available instrument for use in both academic and clinical research. All data collected were handled strictly in accordance with the institution’s data protection policies to ensure the participants remained anonymous from the time of collection through the time of analysis and reporting.

### 2.6. Statistical Analysis

Statistical analysis was conducted using IBM SPSS Statistics version 26. Initial analysis utilized descriptive statistics, including mean (M), standard deviation (SD), and frequency (n), to characterize the sociodemographic profile of the participants. To ensure the integrity of the inferential results, parametric assumptions were rigorously tested prior to analysis. Normality was confirmed through non-significant Shapiro–Wilk tests (*p* > 0.05), skewness within the acceptable range of 1.5 (actual range: −0.42 to 0.55), and a visual inspection of P-P plots. Furthermore, scatterplots of residuals were examined to confirm linearity and homoscedasticity. Potential issues with multicollinearity were ruled out, as Variance Inflation Factors (VIFs) were below 1.45 and the minimum tolerance was 0.72, thereby confirming the independent predictive value of stress and self-efficacy. To evaluate the relationships between the primary variables—stress, self-efficacy, and medication adherence—Pearson correlations were calculated. Following this, a multiple linear regression model was utilized to determine the predictive ability of these variables. Categorical data were transformed via dummy coding to ensure suitability for the regression model; for instance, Gender was represented as Female = 1 and Male = 2. To address the mechanism of these relationships, Hayes’ PROCESS Macro (Model 4) was employed to determine if stress served as a mediator between self-efficacy and adherence. This model was chosen for its robust handling of indirect effects through bootstrap-based standard error estimation (5000 samples) and bias-corrected confidence intervals. This approach provides a more rigorous interpretation of mediation than traditional methods, as it does not rely on the assumption of normality for the sampling distribution of the indirect effect, thereby enhancing the reliability of the study’s conclusions.

## 3. Results

### 3.1. Sample Characteristics

Table 1 presents the socio-demographic of the participants. Of the 270 who participated in the study, the majority of the sample ages 60–70 (43.7%), males (69.3%), and a high percentage of them were married (98.9%). A large proportion were well-educated (61.1%) and a full-time employee (28.1%). The two most common chronic comorbidities of the participants, in addition to heart disease, were diabetes (38.9%) and hypertension (32.2%), and over 1–3 years in terms of the duration of their disease (59.3%).

### 3.2. Descriptive Statistics of Study Variables

Participants reported moderate levels of PSS (mean = 20.17; SD = 3.36) and high levels of self-efficacy in managing their chronic conditions (mean = 44.90; SD = 7.03). The mean ARMS score was 23.38 (SD = 7.06); because higher scores on this scale indicate poorer adherence, this result reflects a tendency toward non-adherence within the sample (Table 2).

### 3.3. Bivariate Correlations

Table 3 presents the correlation between stress, self-efficacy, and medication adherence among the participants. Perceived stress was positively correlated with medication non-adherence (r = 0.392, *p* < 0.01), while self-efficacy was negatively correlated with stress (r = −0.16, *p* = 0.008). Self-efficacy also maintained a significant negative correlation with medication non-adherence (r = −0.332, *p* < 0.01). Given that lower ARMS scores represent higher adherence, this result indicates that increased self-efficacy is strongly associated with better medication adherence.

### 3.4. Predictive Models for Stress and Self-Efficacy

Table 4 presents the factors predicting stress, self-efficacy and medication adherence. The first model (predicting stress) accounted for 18.8% of the variance in stress levels (R^2^ = 0.188, F = 8.69, *p* < 0.001). Gender was the most significant predictor (β = −0.25, *p* < 0.001), followed by Duration of Illness (β = 0.20, *p* = 0.008) and Marital Status (β = 0.11, *p* = 0.045). This suggests that female participants, those with a longer history of illness, and married individuals reported higher stress levels.

Self-efficacy demonstrated the highest explained variance among all models (R^2^ = 0.289, F = 15.19, *p* < 0.001). Age emerged as the strongest negative predictor (β = −0.457, *p* < 0.001), indicating that younger participants maintained significantly higher self-efficacy than older participants. Furthermore, Gender (β = −0.269, *p* < 0.001) and Comorbidities (β = −0.228, *p* = 0.001) were associated with lower self-efficacy. Conversely, a longer Duration of Illness was associated with an increase in self-efficacy (β = 0.278, *p* < 0.00), suggesting a possible adaptation to the condition over time.

The final model investigated factors contributing to medication non-adherence (R^2^ = 0.338, F = 14.22, *p* < 0.001). Crucially, Self-efficacy was found to be the most influential predictor in this model (β = −0.345, *p* < 0.001). The negative coefficient indicates that as self-efficacy increases, rates of non-adherence significantly decrease. Other significant contributors included Gender (β = −0.288, *p* < 0.001), Educational Level (β = −0.249, *p* < 0.001), and Age (β = −0.224, *p* = 0.001). Notably, when accounting for self-efficacy, clinical factors such as comorbidities and duration of illness were no longer significant predictors of non-adherence, highlighting the central role of psychological confidence in treatment adherence.

**Figure 1:**
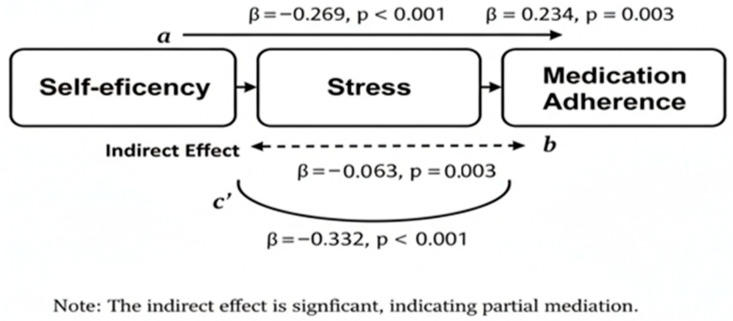
Mediating Effect of Stress Between Self-efficacy and Medication Adherence.

The mediation model illustrates three key relationships among the variables (Figure 1). First, the direct path (c’) shows a significant negative relationship between Self-efficacy and Medication Adherence (β_1_ = −0.332, *p* < 0.001). Second, Path a’ indicates that Self-efficacy has a significant negative effect on Stress (β_2_ = −0.269, *p* < 0.001), meaning higher self-efficacy is associated with lower stress levels. Finally, Path b’ reveals a significant positive relationship between Stress and Medication Adherence (β_3_ = 0.234, *p* = 0.003). Collectively, these paths result in a significant indirect effect (β = −0.063, *p* = 0.003, 95% CI [−0.10, −0.02]). This confirms that Stress serves as a partial mediator; while self-efficacy directly impacts adherence, it also influences adherence by significantly reducing patient stress (Table 5).

The mediation model illustrates three key relationships among the variables (Figure 1). First, the direct path (c’) shows a significant negative relationship between Self-efficacy and Medication adherence (β = −0.332, *p* < 0.001). Second, Path a’ indicates that Self-efficacy has a significant negative effect on Stress (β = −0.269, *p* < 0.001), meaning higher self-efficacy is associated with lower stress levels. Finally, Path b’ reveals a significant positive relationship between Stress and Medication adherence (β = 0.234, *p* = 0.003). Collectively, these paths result in a significant indirect effect (β = −0.063, *p* = 0.003, 95% CI [−0.10, −0.02]).

## 4. Discussion

### 4.1. On Perceived Stress, Cardiac Self-Efficacy, and Medication Adherence

The results of this study provide insight into the psychological factors affecting the management of chronic cardiovascular conditions. This is particularly the case regarding the degree to which clinical outcomes are influenced by both a patient’s outlook on their condition and the medical medication they receive. The relatively high average PSS scores indicate that participants reported feeling a considerable amount of stress. Stress was associated directly with both their self-reported behaviors, as well as to increased risk of adverse cardiac events by patients who are diagnosed with heart failure [21]. Findings from this current research study are consistent with other research study [16]. Previous study had demonstrated link between individuals’ reported levels of stress and decreased adherence to prescribed medications in patients with chronic disease [22]. Due to the fact that moderate levels of stress are prevalent within chronic disease populations, it is clearly necessary to develop interventions that focus on reducing stress and improving a patient’s ability to manage their chronic condition [23].

While the participants exhibited high levels of self-efficacy in their ability to manage their cardiac conditions, the mean adherence score of “moderate” highlights the complex nature of chronic care. While high self-efficacy in this population may indicate better access to resources and education, these individuals likely face fewer barriers to healthcare than those with limited health literacy [24]. The gap between these two values underscores the necessity of providing health education that specifically addresses demographic factors. For example, providing less affluent or less educated populations with targeted education will help ensure that they receive the same level of support as the more affluent or educated populations. Healthcare professionals need to shift away from the traditional medical treatment model and instead incorporate evidence-based structured programs that address both the physical and psychological needs of the individual with a chronic cardiovascular condition. Structured programs allow healthcare providers to help manage stress in ways that have been successful for other populations under similar levels of stress (high-stress). The ultimate goal is to provide improved long-term outcomes for those suffering from heart disease [23].

### 4.2. Correlation of Stress, Self-Efficacy and Medication Adherence

The current research has shown that there is a direct correlation between increased stress and decreased adherence to prescribed medications. As stress increases, so does the lack of adherence to prescribed medications [25]. Health psychology supports this relationship through a body of literature that shows psychological stress negatively affects the ability of individuals to comply with recommended health treatments. There is also a demonstrated negative correlation between self-efficacy, stress and non-adherence to medication. Our study has shown a negative correlation between self-efficacy and medication non adherence. The results show that when patients have confidence in managing their health, they scored less (lower) on the ARMS which reflects better medication adherence. When patients possess the belief in their own ability to manage their own health, they experience less stress and better adherence to their prescribed medication regimen. This is consistent with past research indicating that self-efficacy is a significant psychological construct that promotes healthy behaviors related to managing a variety of health-related conditions including diabetes and hypertension [26,27,28].

Although the present findings are consistent with prior studies concerning the significance of self-efficacy, there have been conflicting results in some studies regarding its influence on adherence. Researchers have concluded that to understand the relationships between self-efficacy and adherence, it is necessary to include factors such as the perception of one’s own health status, the presence of depression, and social support [29]. When patients are self-assured of their ability to control their own health outcomes, they have lower levels of perceived stress. This is more often associated with adherence with prescribed medications for both perceived stress and self-efficacy. Past research has shown that self-efficacy is an important psychological variable that helps influence health-related behaviors [26,27,28], such as disease management for example, diabetes and hypertension. Researchers suggest that perceived stress can influence self-management behaviors in patients with chronic diseases [30,31]. Beyond affecting adherence, stress can also serve as a mediator for interventions designed to improve self-efficacy. More recently, studies have examined the potential mediating effect of perceived stress in the relationship between self-efficacy and medication adherence.

The literature in which these additional variables were included found somewhat inconsistent results concerning a relationship between self-efficacy and adherence. However, the literature has shown a strong association between self-efficacy and better adherence to prescribed medications for a variety of chronic diseases [32]. It is thereby emphasizing the important role self-efficacy plays in both patient education and intervention design [33]. Healthcare professionals may need to routinely assess their patients’ psychological well-being to identify those at risk for high stress or low self-efficacy. Identifying these risks allows providers to offer targeted interventions that effectively lower psychological distress and increase self-efficacy.

### 4.3. Factors Predicting Stress, Self-Efficacy, and Medication Adherence

In addition to gender, the length of time since the onset of an illness, and whether or not a patient is married, also had a statistically significant relationship with levels of perceived stress. Due to the cross-sectional design of this study, we could not determine the exact causal relationship between gender and stress. However, the negative coefficient for gender indicates that women in this population are experiencing higher stress levels than men. These results support past research that demonstrates sex differences in perceptions of and responses to stress. For example, previous research [34] demonstrated that female diabetic patients reported greater levels of stress than male diabetic patients due to a number of psychosocial factors. Likewise, it was suggested that having a chronic disease can result in high levels of stress and anxiety in patients [35].

Research has also established a positive relationship between how long a patient has had their disease and the amount of stress they experience as well. Scholars [35] found that chronic diseases result in high amounts of both stress and anxiety in patients, while researchers [36] noted that due to a perceived lack of effectiveness of medication for chronic diseases, chronic disease patients experience higher levels of stress than others. Conversely, several other variables, which are frequently assumed to influence an individual’s stress levels, were not associated with stress levels in this study. These variables include, but are not limited to: the individual’s age, education level, employment status, and the number of comorbid conditions the individual suffers from. Other studies have demonstrated that each of these variables does contribute to an individual’s stress levels [37,38].

Our results indicated that there was a strong relationship between age and gender and self-efficacy, with the model accounting for approximately 28.9% of the total variance in self-efficacy. Therefore, both younger age and being female were associated with lower self-efficacy. Our finding of a negative association of younger age and self-efficacy is interesting given previous literature indicating that younger people generally have higher self-confidence. Nonetheless, it was found that older people, although they often face more health problems, are able to manage their chronic health conditions using effective coping strategies and exhibit greater self-efficacy [39].

We also found that longer durations of illness were positively associated with self-efficacy scores, which is consistent with other research showing that patients with chronic conditions develop greater self-efficacy through repeated experiences with managing their condition [40]. Furthermore, our finding that having comorbidities is negatively associated with self-efficacy is also consistent with previous research indicating that patients with multiple comorbidities experience difficulty with feeling confident in their ability to manage their health [41]. Consistent with previous research, our results also indicate that gender continues to play a significant role in the self-efficacy of patients with chronic conditions, with females exhibiting lower self-efficacy than males [34].

According to the regression model results, several predictor variables are significantly related to the level of medication adherence. Gender and education levels were found to be two of these significant predictor variables. A number of studies have shown education to be a highly predictive variable in terms of medication adherence [35]. Researchers specifically identify education among the highest predictors of an individual’s adherence to medication [35]. Education is beneficial to the patient by providing the information required for proper use of medications and by assisting patients in understanding their health condition and medication options. Consistent with prior research [35], age was an additional factor that influenced adherence with medication regimens. Older adults were shown to be significantly more likely to comply with medication regimens based upon the development of routines for their healthcare. In contrast to prior research [38], it showed that older adults are more likely to comply with medication regimens than younger adults. This is because they have a greater awareness of their healthcare needs. No relationship between marital status, employment status, comorbid conditions, or length of illness and adherence with medication was observed in this study. While there is evidence from prior research indicating that each of these factors can influence adherence behaviors [35,37], the absence of a correlation in this study demonstrates the complexity of factors that influence adherence to medication and supports continued exploration into the heterogeneity of factors that affect adherence.

### 4.4. On the Mediating Effect of Stress

Stress was a link to the connection of participants’ self-efficacy and adherence, in accordance with our hypothesis. It can be seen that self-efficacy acts not only as a direct motivator for a patient’s behavior, but also as a means to alleviate the psychological burden of stress. Self-efficacy is demonstrated to mediate the relationship between depressive symptomatology and adherence to prescribed antihypertensive medications [41]. This indicates that a person’s confidence in their ability to manage their condition is a significant factor in adherence, regardless of the level of depression or other negative emotional state experienced by the individual. In addition, it was demonstrated that older Korean patients who were hypertensive adhered to their medications at a higher rate when they had a high degree of self-efficacy [42]. This further demonstrates the important role of self-efficacy in managing chronic health conditions.

The findings also supported an additional statistically significant indirect pathway that shows stress may be able to disrupt the relationship between self-efficacy and medication adherence. These results indicate a need to address several issues related to non-adherence (such as self-efficacy and stress) in order to comprehensively treat patients who have chronic illness. Results of this study support the development of interventions that are intended to increase adherence to prescribed medications; such interventions should contain elements that will increase self-efficacy and reduce stress levels to achieve maximum adherence to medication recommendations. Additionally, interventions focused on enhancing self-efficacy have been found to decrease stress and, therefore, create an environment where individuals are able to obtain better adherence [43]. Research has shown that self-efficacy is a mediator in the relationship between medication literacy and adherence [43], providing evidence to support education-based interventions to increase adherence. As such, an integrated approach to self-management that addresses both self-efficacy and stress management is essential to improving patient outcomes [44]. This emphasizes the importance of developing both self-efficacy and effective stress management skills. While the present findings highlight the importance of self-efficacy and stress management in adherence to medications, future research could investigate the interaction of these two factors and how stress reduction methods, such as mindfulness practices, can promote self-efficacy and lead to long-term adherence behaviors. Additional areas of investigation for future studies could include investigating the effects of self-efficacy and stress on medication adherence across various types of chronic illnesses and developing targeted interventions for each type of patient population.

### 4.5. Study Implications

The findings of this study suggest that improving medication adherence (ARMS) requires moving beyond a purely educational model toward a multidimensional, psychosocial framework. Below are the specific ways this should be operationalized across the healthcare spectrum. Since self-efficacy (CSES) was identified as a direct determinant of adherence and a buffer against stress, clinical interactions must prioritize psychological empowerment over simple information delivery. Instead of traditional lecturing, nurses should utilize “Teach-Back” methods focused on self-management. For example, a nurse should ask a patient to simulate how they would manage their medication schedule during a high-stress event (e.g., a family emergency), thereby building the patient’s mastery of their own routine. Also, Motivational Interviewing (MI) should be standardized in consultations. Physicians should assess a patient’s “Confidence Score” (based on CSES principles) on a scale of 1–10. Patients scoring below 7 should be flagged for immediate behavioral intervention or a follow-up call from a clinical pharmacist.

Because stress (PSS) was found to be a major mediator of non-adherence, administrators must integrate psychological health into the physical health workflow. Healthcare organizations should integrate the PSS-4 (short form) into the Electronic Health Record (EHR) as a standard vital sign. Moreover, policy should dictate that a PSS score indicating “High Stress” triggers an automatic referral to a social worker or counselor. This prevents the “decline in adherence” identified in the study before it occurs. On the other hand, the statistical vulnerability of younger males with lower education levels confirms that “one-size-fits-all” education is a policy failure. For healthcare organizations, they should develop mHealth (mobile health) platforms that use visual-heavy, low-literacy content and SMS “nudges” rather than text-heavy pamphlets. Moreover, programs should be designed to use “plain language” and peer-led support groups where younger men can observe successful medication management modeled by their peers, which is a key driver of self-efficacy as consideration for gender-sensitive support.

### 4.6. Study Limitations

Limitations in this study need to be recognized. One limitation of the study is that it uses a cross-sectional design that involves a single data collection point in time and, therefore, does not allow for the identification of a direct relationship between stress, self-efficacy, and the patient’s level of medication adherence. The study’s demographics present challenges to the representativeness of the study population. Most of the participants were married males (69.3% male; 98.9% married). The unbalanced demographics can allude to a “healthy volunteer” effect, i.e., study participants were recruited through a pipeline that was too narrow and/or biased to men with the adequate socioeconomic means to take care of their health. Moreover, the fact that almost all study participants were married suggests that they have more social support and caregiving readily available to them, unlike the more than three quarters of the diabetic (38.9%), hypertensive (32.2%), and possibly other populations. This implies that the results cannot be extended to women or to single people who face different psychological hurdles to controlling their diseases. In subsequent studies, researchers must utilize stratified sampling techniques to sufficiently differentially represent the sample with respect to gender and marital status to validate the findings among varied social settings.

A further study limitation is the generalizability of the study, due to the use of a convenience sample of heart failure patients and the single hospital site (Aseer Central Hospital). Although this facility represents a significant tertiary hospital in Saudi Arabia, the study findings may not accurately represent the experiences of heart failure patients living in rural areas or receiving care from primary care physicians. Furthermore, although the Cronbach’s alpha coefficients for the PSS (0.83), CSES (0.80), and ARMS (0.78) demonstrated good internal consistency reliability, they were calculated using a pilot study of only 20 subjects. Therefore, it is possible that the low sample size used to test the preliminary reliability of each instrument does not adequately represent the psychometric properties of the three measures with respect to a larger, potentially more heterogeneous population. Consequently, more comprehensive and rigorous validation research should be conducted in subsequent investigations. In addition, since no cognitively impaired patients were included in the study, nor were critically ill patients, the study results apply best to a relatively stable subgroup of the NYHA Class II-IV population. Lastly, researchers should take caution when evaluating the results of the ARMS because the reverse scoring method used in the ARMS (i.e., lower numbers indicating greater adherence) differs from the forward scoring methods used in both the PSS and the CSES, and therefore requires careful consideration throughout the correlational and regression analyses.

## 5. Conclusions

The findings illustrate an interaction among psychological resources and medication outcomes in which the most at risk are identified by demographic factors (younger, less educated, males). The results show self-efficacy to be the most important internal protective factor. Self-efficacy acts as a significant predictor of medication adherence, with perceived stress serving as a partial mediator in this relationship. Thus, the results suggest that clinical interventions need to focus on developing a patient’s confidence in managing their care, rather than simply educating them, to develop a long-term plan that ensures that they will continue to take their medications as prescribed.

## Figures and Tables

**Table 1:** Socio-demographic characteristics of the participants. N = 270.

Variable	Category	Frequency (n)	Percentage (%)
Age (years)	34–40	17	6.3
	41–59	111	41.1
	60–70	118	43.7
	71–77	24	8.9
Gender	Male	187	69.3
	Female	83	30.7
Marital status	Married	267	98.9
	Unmarried	3	1.1
Educational level	Preparatory	13	4.8
	Primary	22	8.1
	Secondary	48	17.8
	High School	22	8.1
	Higher Education	165	61.1
Employment status	Full Time Employee	76	28.1
	Part Time Employee	63	23.3
	Retired	75	27.8
	Unemployed	56	20.7
Comorbidities	Hypertension	87	32.2
	Diabetes	105	38.9
	Lung Diseases	22	8.1
	Obesity	56	20.7
Duration of illness (years)	<1	63	23.3
	1–3	160	59.3
	4–6	29	10.7
	>6	18	6.7

**Table 2:** Descriptive statistics of study variables.

Variable (Scale)	Max Possible	Min Score	Max Score	Mean (M)	SD
Perceived Stress (PSS)	40	12	25	20.17	3.36
Cardiac Self-Efficacy (CSES)	52	26	52	44.9	7.03
Medication Adherence (ARMS)	48	12	43	23.38	7.06

**Table 3:** Correlation between stress, self-efficacy and medication adherence among participants.

Variables	1. Stress	2. Self-Efficacy	3. Med. Adherence
1. Stress	—		
2. Self-efficacy	r = −0.160, *p* = 0.008	—	
3. Med. Adherence	r = 0.392, *p* = 0.001	r = −0.332, *p* = 0.001	—

**Table 4:** Factors predicting stress, self-efficacy and medication adherence.

Variables	R	R^2^	F	B	SE	β	t	*p*
Predicting Stress	0.434	0.188	8.69 *					
Constant				16.99	2.51	—	6.78	0.001
Age				0.58	0.3	0.13	1.92	0.059
Gender				−1.78	0.49	−0.25	−3.66	<0.001
Marital status				3.61	1.8	0.11	2.01	0.045
Educational level				−0.21	0.19	−0.08	−1.13	0.259
Employment status				−0.12	0.19	−0.04	−0.6	0.548
Comorbidities				−0.08	0.13	−0.05	−0.61	0.541
Duration of illness				0.87	0.33	0.2	2.67	0.008
Predicting Self-Efficacy	0.537	0.289	15.19 *					
Constant				52.418	4.907	—	10.683	<0.001
Age				−4.322	0.589	−0.457	−7.341	0.001
Gender				−4.094	0.951	−0.269	−4.306	0.001
Marital status				6.239	3.518	0.093	1.774	0.077
Educational level				−0.377	0.363	−0.067	−1.039	0.3
Employment status				0.567	0.38	0.089	1.494	0.136
Comorbidities				−0.854	0.257	−0.228	−3.317	0.001
Duration of illness				2.5	0.64	0.278	3.905	0.001
Predicting Medication Non-Adherence	0.581	0.338	14.22 *					
Constant				26.269	5.066	—	5.185	<0.001
Age				−2.127	0.608	−0.224	−3.5	0.001
Gender				−4.401	0.982	−0.288	−4.482	<0.001
Marital status				−5.418	3.632	−0.081	−1.492	0.137
Educational level				−1.419	0.374	−0.249	−3.79	<0.001
Employment status				−0.644	0.392	−0.101	−1.643	0.102
Comorbidities				−0.267	0.266	−0.071	−1.003	0.317
Duration of illness				−0.336	0.661	−0.037	−0.509	0.611
Self-efficacy				−0.412	0.085	−0.345	−4.847	<0.001

Legend: R (Correlation Coefficient); R^2^ (Coefficient of Determination); F (F-statistic); B (Unstandardized Coefficient); SE (Standard Error); β (Beta/Standardized Coefficient); t (t-statistic); *p* (*p*-value)* Significant at *p* < 0.001.

**Table 5:** Mediating Effect of Stress.

Path	Relationship	β	SE	t	*p*-Value	95% CI
Direct (c’)	Self-efficacy → Med. Adherence	−0.332	0.073	−4.55	<0.001	−0.48 to −0.19
Indirect	Self-efficacy → Stress → Med. Adherence	−0.063	0.021	−3	0.003	−0.10 to −0.02
Total	Self-efficacy → Med. Adherence	−0.395	0.068	−5.81	<0.001	−0.53 to −0.26

Note: The R^2^ values ranging from 0.188 to 0.338 indicate that while the study successfully identified significant psychological predictors, medication adherence in cardiology patients is multi-factorial. These moderate values highlight that future research should incorporate environmental and economic variables, such as medication costs and social support systems, to further explain the remaining variance.

## Data Availability

The datasets presented in this article are not readily available because they contain sensitive participant information and are subject to ethical restrictions approved by the King Khalid University Ethical Committee.
